# Voice Strain in Teachers

**Published:** 1902-05-10

**Authors:** J. E. McDougall

**Affiliations:** Hon. Assistant Laryngologist Liverpool Stanley Hospital.


### Hospital Clinics and Medical Progress.

### VOICE STRAIN IN TEACHERS.

By J. E. McDougall, M.B.Vict., M.R.C.S., Hon. Assistant Laryngologist Liverpool Stanley Hospital.

The laryngologist cannot but be struck by the number of teachers from elementary schools of all denominations who present themselves from time to time for special treatment. Naturally this is caused to some extent by excessive use of the voice in an oftentimes impure atmosphere. One sees this at once on observing the conditions under which school teachers work; the class-room sometimes crowded with children whose attention one often fails to retain by even the most interesting presentation of mental pabulum.

There is, further, the very objectionable practice of teaching several classes in the same room which results in a "shouting" competition between the various teachers, and places an unwholesome strain on the voice. This is one of the several remediable causes of laryngeal difficulties which call for treatment.

Another source of trouble is the ignorance of voice-production, which prevails so largely amongst teachers and public speakers generally; an ignorance which is very discreditable to all concerned. When we consider that no pains are taken to train, develop and perfect an organ on which speakers and teachers must depend to convey their message and practise their vocation, we must wish that voice-production could form an important part of the curriculum of the teacher, even though some of the fancy subjects that worry him went by the board.

I am quite sure that even with a good method of voice-production and the exercise of his calling under suitable conditions, fatigue of the voice in the subject of an indifferent constitution produces a good deal of disturbance, and pre-disposes to laryngeal trouble. This must be enormously increased in those more unfavourably placed, who have used no intelligent method of voice-culture and who habitually abuse the voice.

But there are other considerations than those of bad habits and defective environment. Bad as these are in the otherwise healthy, what shall we say to the subject of disease—those candidates for the teaching profession who are undoubtedly physically unfit, whose certificate of health has overlooked the very important consideration of the health and construction of the vocal organs and respiratory passages? Certificates given at the outset of the candidates' career should not be accepted unless they imply a careful and competent examination of the throat and nose. There are undoubtedly many candidates who, from such causes as old adenoids, thickened pillars of the fauces, stenosis of the nasal passages, hypertrophic rhinitis, etc., are quite unfitted to engage in teaching before they have undergone suitable treatment. Even then it is in my experience that some of these patients cannot be sufficiently improved to withstand the strain of constant speaking, more especially under such adverse conditions as have already been commented upon. Such candidates should be excluded at the outset of their career rather than permitted to continue their course of training until constantly recurring loss of voice compels them to seek their livelihood in other directions.

The candidate then should undergo as part of his or her training a course of voice-production under some properly accredited tutor. For it cannot be doubted that it is occasionally less in the use, and oftentimes more in the abuse of the voice, that the loss of the latter is effected.

Lastly, each class should be taught in a separate room. Very few voices, however perfect initially, can endure the strain of competing for a hearing above the din of several classes, and as many teachers, congregated in one large room. Such aggregation is not fair to the teacher, it is unfair also to the pupils. The one is overstrained and cannot give of his or her best, and the children often have to receive on their bodies what the teacher, having lost in patience and enthusiasm, is unable to impress upon their minds.

